# A qualitative analysis of Chinese novice general dentists’ perspectives on using ergonomic loupes in clinical practice

**DOI:** 10.1371/journal.pone.0353061

**Published:** 2026-07-09

**Authors:** Kexian Xie, Yuangao Li, Xiao Wang, Ning Ma

**Affiliations:** Department of Stomatology, Peking University Third Hospital, Beijing, PR China; PGIMER: Post Graduate Institute of Medical Education and Research, INDIA

## Abstract

**Background:**

Ergonomic loupes are the next-generation loupes with built-in refractive prisms that enable the user to sit up straight, look straight ahead, and reduce neck tilt while viewing the operating field from down below. They differ greatly from traditional loupes in optic design and viewer perspective. They appeared on the market in 2022 and are quickly gaining popularity in China. In this study, we sought to use semi-structured interviews and thematic analysis to assess the perspectives of novice Chinese general dentists on using ergonomic loupes in clinical practice.

**Methods:**

The study population consisted of nineteen general dentistry residents undergoing training at Peking University Third Hospital in Beijing, China. Semi-structured interviews were conducted individually with participants. The interview data were analyzed thematically using NVivo 11 software.

**Results:**

The findings revealed that the benefits of using ergonomic loupes included enhanced visual acuity, more precision, improved posture, and improved treatment quality. Main disadvantages included discomfort from use, splash protection issue, fixed viewpoint, and limited field of view. There was typically a brief adaptation process for the new user. Participants were weighing cost and benefit in deciding whether and where to use ergonomic loupes. Comparative experience showed that ergonomic loupes had ergonomic advantages, while traditional loupes had hand-eye coordination advantages.

**Conclusion:**

The participants had overall positive perspectives on using ergonomic loupes in clinical practice. Comparative experience showed that ergonomic loupes had ergonomic advantages, while traditional loupes had hand-eye coordination advantages.

## Introduction

Dentistry is considered a physically and mentally demanding profession. The limited operating field of the oral cavity makes it a challenge to obtain an unobstructed clear view while maintaining neutral body posture. Dental practitioners tend to lean in close and adopt an unbalanced posture to gain a better view of the operating field [1,2]. Also, the dental practitioner has to maintain a static posture for prolonged periods *(i.e.,* the mouth mirror arm, the head and neck, and the torso) and perform repetitive movements (*i.e.,* the operating arm) at the same time. As a consequence, musculoskeletal disorders (MSDs) are common among dental practitioners [3–5]. Commonly reported symptoms of MSD in dental practitioners include shoulder, back, and neck pain [3]. Severe forms of MSD can affect productivity, the quality of life, and disrupt the working schedule of the affected dental personnel [6,7].

The use of magnification (loupes and dental operating microscope) has been shown to improve the dental practitioner’s visual acuity [8–11], body posture [12–14] and quality of treatment procedures [15,16]. Compared with dental operating microscope, loupes are considered more comfortable and easier to use but offer less ergonomic improvement [14]. Traditional loupes can be classified as either Galilean or Keplerian/Prismatic loupes. The Galilean loupes are lighter and cheaper but generally have lower magnification power. The prismatic loupes, due to their sophisticated optic design, are heavier and more expensive but have higher magnification power [17]. In recent years, there has been a trend of implementing loupes in undergraduate and postgraduate training in dental schools [18]. The benefit of early adoption of magnification in training is that the students are encouraged to develop better posture while performing treatments. This could help to prevent or delay the onset of MSDs in their future career [19]. Furthermore, perceived benefit of using loupes on treatment quality can increase the clinicians’ confidence in providing high standard care for patients [20].

In mainland China, young dentists are required to finish a 5-year residency program before they can work in a public hospital. Unfortunately, few dental schools in mainland China implement loupes in training [21]. Few students purchase loupes and use them in practice. As a result, most residents are not familiar with loupes. Implementing loupes in general dentistry residency training may benefit the trainees throughout their careers by improving body posture and treatment standards.

Ergonomic loupes are the next-generation loupes with built-in refractive prisms that enable the user to sit up straight, look straight ahead, and reduce neck tilt while viewing the operating field from down below [22]. They differ greatly from traditional loupes in optic design and viewer perspective. They appeared on the market in 2022 and are quickly gaining popularity in China (*e.g.,* DFK surgical loupes by Zumax, Vision Up Ergonomics loupes by Design for Vision, Ergo loupes by Orascoptics, ErgoPrism Vario loupes by Lumadent, *etc.*). Although there has been a qualitative study of user perspective on dental magnification loupes [18], the magnification loupes in question were conventional Galilean type loupes. Because ergonomic loupes are relatively new on the market, there is no qualitative study on their user experience yet. A study on user perspective is needed because it can provide future reference for prospective users and help manufacturers to improve their products.

Most qualitative research publications in health care describe the use of interviews and focus groups [23]. The thematic analysis method is widely used in qualitative health care studies [24–28].

This study aimed to use semi-structured interviews and thematic analysis to assess the perspectives of general dentistry residents on ergonomic loupe usage in clinical practice.

## Materials and methods

This qualitative study was reported in accordance with the principles of the Consolidated Criteria for Reporting Qualitative Research (COREQ) [23]. This study was approved by the Ethics Committee of Peking University Third Hospital (IRB00006761-M2024937). Written informed consent forms were obtained from all study participants.

### Participant recruitment

Purposive sampling was used for recruiting participants. We sought to increase the diversity of our study sample by including participants of both genders at various stages of training, and with various educational and specialty backgrounds. General dentistry residents undergoing training (novice dentists) in the dental department of Peking University Third Hospital in Beijing, China were invited to take part in the study. Recruitment continued until data saturation was reached (no new codes were generated), as determined by the principal investigator. In total, twenty-three general dentistry residents were recruited. Recruitment started on 14/01/2025 and ended on 15/02/2025. Four of the invited residents declined to participate in the study because of time constraints. Four participated in the pilot interviews, and nineteen participants were included in the final analysis. They were affiliated with four regional general hospitals located in different districts of Beijing. All participants had used ergonomic loupes (DFK surgical loupes, Zumax, China) and traditional loupes (SLE surgical loupes, Zumax, China) in the clinical setting for six months before the interviews. Both types of loupes were provided through the residency program and shared among residents. After the study, the residents could opt to buy their own loupes. The demographics of the nineteen participants are shown in Table 1.

**Table 1:** Demographics of participants with respect to gender, age, dental education degree, specialty background, and year of residency.

Gender	
Male	6
Female	13
Age, years	
<30	11
30-40	8
Dental education degree	
Bachelor	3
Master	5
Doctor	11
Specialty background	
General dentistry	3
Oral and maxillofacial surgery	1
Orthodontics	1
Endodontics	4
Prosthodontics	4
Periodontics	6
Year of residency program	
2nd year	3
3rd year	8
4th year	2
5th year	6

### Data collection

Semi-structured face-to-face interviews regarding user experience of ergonomic loupes were conducted individually with participants in a quiet room without the presence of non-participants. One researcher (KXX), an experienced male general dentist (DDS) who had undergone substantial training in interview skills and qualitative analysis, conducted the interviews in Chinese. The interviewer was not involved as an instructor in the departments’ training program, so there was no hierarchical relationship between the interviewer and the participants, which may affect the openness of the interview. The interviews were conducted around a list of in-depth open-ended questions as an interview guide. The participants were encouraged to freely communicate their experience of ergonomic loupe usage and express their views on the topic. The draft interview questions were devised after studying themes of previous published qualitative research on dental loupes [18]. Each interview lasted around 30 minutes. Before the study, the interviewer (KXX) conducted pilot interviews with four participants to refine the question lists. The interview transcripts were reviewed by three clinician experts outside the research team. The participants’ and experts’ comments were collected and analyzed for face and content validity of the interview questions. The pilot interviews were excluded from the final study. The refined interview questions were centered around topics as follows:

1. Adaptation process and time required;
2. Symptoms associated with ergonomic loupe usage;
3. Effects on posture, visual acuity, and quality of treatment;
4. Advantages and disadvantages;
5. Usage scenario;
6. How are ergonomic loupes different from traditional loupes?

### Data analysis

The interviews were recorded with the interviewer’s smartphone. The recordings were transcribed verbatim with the help of the Microsoft Voice Input system. The accuracy of the transcripts was verified by two researchers (YGL and NM). The verified transcripts were returned to participants for comments and correction. The thematic analysis method was used to analyze the interview transcripts. The analysis included six phases, as described by Braun and Clarke [27]: 1. familiarizing with the data; 2. generating initial codes; 3. searching for themes; 4. reviewing themes; 5. defining and naming themes; and 6. producing the report. Theanalysis was performed using NVivo 11 qualitative analysis software as a support. Initial line-by-line coding and candidate theme development were carried out individually by all four researchers. Following initial coding, researchers met to discuss and finalize coding and themes. All researchers checked the final codes and themes against the data. The findings of the analysis underwent member checking by 10 participants to ensure the trustworthiness of the analysis.

## Results

Five themes and twenty-two sub-themes were identified after the analysis process. The five themes were: advantages of using ergonomic loupes, disadvantages of using ergonomic loupes, adaptation process, why use ergonomic loupes, and how they are different from traditional loupes. Salient quotations from the interview transcripts were selected to illustrate each theme. Participant identification was represented by a numeric code in parenthesis after each quotation. A summary of themes and sub-themes is listed in Table 2.

**Table 2:** Summary of themes and sub-themes in this study.

Themes	Sub-themes
Advantages of using ergonomic loupes	Minimally invasive
	Better treatment results
	Enhanced visual acuity
	Improved posture
	Better control of fine movements
Disadvantages of using ergonomic loupes	Limited field of view
	Switching field of view
	Loupe vibration
	Fogging
	Lack of splash protection
	User-friendly issue
	Discomfort from use
	Fixed viewpoint
Adaptation process	Hand-eye coordination
	Working distance determination
	Finding the field of view
	Adaptation time
Why use ergonomic loupes	Usage scenario
	Cost consideration
	Weighing cost and benefit
How are they different from traditional loupes	Better posture
	Less coordinated hand movement

### Advantages of using ergonomic loupes

Participants found working with ergonomic loupes tends to be minimally invasive.

“…and is more conservative…” (15). Some stated using ergonomic loupes could produce better treatment results. “…my treatment results are great owing to it…” (1), “…my scaling is better when I use ergonomic loupes…” (13). Many participants expressed that they had enhanced visual acuity when using ergonomic loupes. “…I can see my cavity preparation more clearly…” (10).

All participants mentioned that they had better posture when using ergonomic loupes, which reduced the onset of musculoskeletal disorders.

“…I sit straight and my neck is more comfortable…” (8), “…I used to have neck pain, now I don’t…” (12), “...It is definitely an ergonomic breakthrough, you don’t have to lower your head at all, you just look straight ahead, and you don’t have neck and back pain…” (1).

Participants found using ergonomic loupes offer better control of fine movements.

“…I have better control of fine movements…and my preparations are better when I use the ergonomic loupes…” (8)

### Disadvantages of using ergonomic loupes

Some participants complained the ergonomic loupes had limited field of view.

“…the field of view is too small…” (11).

Some participants complained that she had to frequently switch between using peripheral vision to pick up instruments outside the operating field and looking through the loupes to see the tooth.

“…the switching was too often to get used to…” (11).

Some participants reported vibration of the loupes caused by sudden movement or physical tremors of the head.

“…you move slightly, because it is magnified, the field of view vibrates…” (12). Some participants mentioned fogging could be troublesome in cold weather. “…in winter, the glasses got foggy, because the water vapor got through the surgical mask and contacted the lens and caused fogging…” (1).

Some participants found splash protection a problem, while others found it less important.

“…it does not have protective glasses to shield your eyes. Even when you are wearing goggles, because you are looking straight ahead, the bottom of the mask is open, so particles and liquids can still get into your eyes. Once, my right eye got contaminated by a patient’s saliva. I didn’t rinse my eyes immediately. The patient had a little cough. The next day, my eyes got all red. I was diagnosed with viral conjunctivitis, which later developed into viral keratitis. My condition healed eventually after a month, leaving corneal scars. Luckily, my eyesight was not affected…” (2), “… splash contamination can be a problem” (14), “…if you are far enough from the patients, splash contamination (of the eyes) can be avoided…” (7). Some users with myopia had reduced working distance when they were using the loupes. “…because I have severe myopia, I had to lean very close to the patient…” (4).

Using ergonomic loupes for prolonged duration can cause discomfort. Commonly reported discomforts after prolonged use included eye strain, headache, and dizziness. Complaints about the heavy frame pinching the nose or ears were also reported.

“…my eyes were a little dry…” (10), “…when I got back from work, my eyes were red and dry, sometimes my extraocular muscles were painful, sometimes I couldn’t see clearly after taking off the loupe…” (1), “…it was a little heavy, my ears got painful…” (9), “…it was pinching my nose, you can see the imprint on my nose…” (3), “… I felt some dizziness…” (12), “…my head hurts after some time…” (5)

When using the ergonomic loupe, the viewpoint of the user is relatively fixed, it might be difficult for the user to change viewpoint during procedures.

“...It was not versatile enough, especially when you are doing surgery. When you are scaling, your posture is somewhat fixed, it is OK to use ergonomic loupes. But when you are doing surgery, you may want to change your viewpoint, and the ergonomic loupe may become troublesome...” (19)

### Adaptation process

The ergonomic loupes may require some time for the users to adapt to them. Many participants reported hand-eye coordination problems when they started to use ergonomic loupes.

“…When I first put it on, it was strange, it was like my brain didn’t know where my hands were. It took me several minutes to get used to it…” (17), “…you were moving in the wrong direction, it was a little confusing at first…” (16), “…I had trouble controlling the depth of my preparation, the cavity I prepared (using an ergonomic loupe) was too shallow…”(9), “…the viewing angle was strange at first, but after some practice, I got used to it…” (16).

It was difficult for some participants to find the right working length and field of view.

“…it was hard to find the right working distance…” (8), “…I found it hard to use at first because it was difficult to find the field of view…” (10). However, the adaptation time was typically short. “…it was easy to use” (8), “…I got used to it after one or two cases” (10)

### Why use ergonomic loupes

The users might choose specific clinical scenarios to use ergonomic loupes. Some participants reported using the ergonomic loupes only in procedures requiring fine motor control, while some reported using the loupes in every procedure.

“…I use it routinely, I even use it to find second mesio-buccal canals in maxillary molars…” (14), “...I use it for root canal treatment...” (18), “…I don’t use it for tooth extractions because of blood splash contamination…” (17), “…I use it for posterior teeth and when doing prosthetics…” (7).

Some mentioned that cost was the main factor to consider when choosing ergonomic loupes. “…I chose it not for any other reason, just because it was the cheapest, others were too expensive…” (13).

The users were weighing cost and benefit in deciding whether to use ergonomic loupes in clinical practice.

“…my neck was so painful, I had to use it, even though it causes eye strain, pinches my nose, or else I would have a cervical spine surgery in a few years if my neck condition worsened…” (1)

### How are they different from traditional loupes

Compared with traditional loupes, ergonomic loupes had advantages in ergonomics.

“...compared to the traditional loupe, I don’t have to lower my head anymore, my waist is much better too. It is really an ergonomic breakthrough...” (18)

“...My posture is definitely better now than before, when I was using my Galilean type traditional loupes...” (19)

Compared with traditional loupes, the hand-eye coordination may be different for ergonomic loupes.

“...Because it is an ergonomic loupe, my hands may not be where I am looking at. It feels different. ” (19)

## Discussion

The benefits of using traditional loupes include improved visual acuity [8,29], better posture [8,12–14,30,31], increased precision [32,33], and decreased MSD symptoms [34]. However, the improvement in procedure quality by using loupes was somewhat controversial [12,16,35–38]. Murbay *et al.* used a digital approach to compare the quality of crown preparation performed by students with and without loupes and found that the quality was not affected by the use of loupes [35]. Contradicting results were given by Braga et al. In their study, preparations marks and pass rate (endodontic access cavity and crown preparation) were better using magnification [16].

In our study, enhanced visual acuity, better posture, improved precision, and better treatment results were also associated with ergonomic loupe usage by participants. However, these perceptions were not objectively measured clinical outcomes but perceived improvements, as our study design (qualitative analysis) did not directly assess clinical performance. Whether ergonomic loupes out-perform traditional loupes in the above-mentioned aspects should be further studied in quantitative studies.

In our study, most reported disadvantages of ergonomic loupes (*i.e.*, limited field of view, switching field of view, loupe vibration, fogging, discomfort, fixed viewpoint, *etc.*) were related to user experience. The splash protection issue, however, warrants special attention because it may increase the occupational exposure risk of the user. The ergonomic loupe is designed so that the user can look straight ahead while viewing the operating field from down below. This means the user may have the unshielded bottom of the loupe/face shield facing the operating field. Protection from blood/saliva splash contamination represents a serious issue in dentistry [39]. Further modification of loupe design or other measures are needed to address this problem. Specially designed face shields that cover the chin or through-the-lens design ergonomic loupes with large shielding glasses may better protect the user from splash contamination.

The new “look straight ahead” design of ergonomic loupes can be difficult to adapt to at first, because dental practitioners are trained to look downward at the operating field with their naked eyes or through traditional loupes. In our study, there was some confusion in finding the operating field for the new user, and hand-eye coordination with ergonomic loupes also required some time to improve. But the adaptation time was not long for most users. This is perhaps because our participants were young and were more adaptable to new things than senior dental practitioners.

The most commonly reported areas of traditional loupe usage are restorative dentistry, dental surgeries, endodontics, and prosthodontics [20,34]. In this study, there were preferred usage scenarios for ergonomic loupes too. Cost was considered a negative factor or a barrier to using traditional loupes [20,34]. This remains true for ergonomic loupes in this study. The cost could be deterring for young general dentistry residents who have just come out of school.

In this study, it was found that the users were weighing the cost and benefit of using ergonomic loupes in clinical practice. The practitioners have to decide whether, where, and when to use the ergonomic loupes, after weighing the benefits (*e.g.*, improved visual acuity, better posture, less MSD symptoms, increased precision, *etc*.) and the disadvantages (*e.g.*, additional expenses and discomforts). The practitioners should be well informed of the benefits of using ergonomic loupes before making decisions. The sales representatives and senior dental practitioners may be the sources of such information.

In our study, participants’ comparative experiences of using ergonomic loupes and traditional loupes indicated that ergonomic loupes had an ergonomic advantage, although they were associated with less coordinated hand movement. However, the comparative experiences derived from this study are limited. To further investigate this subject, quantitative studies are warranted in the future.

The ergonomic loupes used in our study were of the same model (DFK surgical loupes, Zumax). Since different ergonomic loupe systems vary in magnification, working distance, and optical design, the findings may not necessarily apply to all ergonomic loupe systems. This is another limitation of our study.

## Limitations and future directions

To our knowledge, this is the first qualitative study on ergonomic loupe usage in clinical practice. However, our study had some limitations. Qualitative studies cannot provide objective measurement of the comparative performance of ergonomic loupes and traditional loupes. Future quantitative studies are warranted. The participants in our study were novice dentists. As experience is a confounding factor in loupe studies [9].

Inclusion of objectively measured clinical outcomes with control of the confounding factors to look for improvement resulting from using the ergonomic loupes compared to the traditional loupes may be considered in future studies. The confounding factors may be the duration of usage of the two types of loupes, the nature of the clinical cases dealt with, etc.

Modification of loupe design or other measures may be explored in further studies to address the problem of possible contamination resulting from blood/saliva splash while using the ergonomic loupes.

The cost-benefit analysis of using different types of ergonomic loupes may be considered in future research.

As experience is a confounding factor in loupe studies, studies should include experienced dentists to validate the results of our study.

## Conclusion

The participants had overall positive perspectives on using ergonomic loupes in clinical practice. Comparative experience showed that ergonomic loupes had ergonomic advantages, while traditional loupes had hand-eye coordination advantages.
